# Dyadic Resistance in Parent‐Adolescent Interactions During the Transition to High School

**DOI:** 10.1002/jad.12451

**Published:** 2024-12-15

**Authors:** Daniel Ji, Sheila K. Marshall, Grant Charles

**Affiliations:** ^1^ School of Social Work, Faculty of Arts The University of British Columbia Vancouver British Columbia Canada

**Keywords:** disagreement, dyadic, parent‐adolescent conflict, parent‐adolescent resistance

## Abstract

**Introduction:**

Although prior research has examined adolescents' resistance to parental control, the dyadic level of analysis has been overlooked. This study attended to how a Canadian sample of parents and adolescents engaged in resisting one another by observing moment‐to‐moment actions as they discussed the upcoming transition to high school.

**Methods:**

A secondary analysis of data collected from 2010 to 2012 using the Action‐Project Method was conducted. The sample of 27 parent‐adolescent dyads (23 mothers; 4 fathers); 13 boys, 14 girls (Mean youth age = 13.3) was recruited from two urban centers. Videorecorded self‐directed conversations were immediately followed by open‐ended video recall interviews. A novel way of analyzing data at the dyadic level of analysis was developed based on critical reflexive thematic analysis guided by social constructionist theory.

**Results:**

Resistance was observed at least once in 23 of the 27 conversations (total = 97, range = 0−9, Mean = 3.63, SD = 2.69). Four distinct themes were developed: *Reminding of and then defending the “constant battle” lines*, *Cautious avoidance*, *the Nudging match*, and *No point anymore and minimal responses*. Dyads varied in frequency and number of themes in their conversations with 78.56% engaging in more than one theme. Most differences came to a trickling of resistance wherein members did not agree but continued to interact without extending the resistance further.

**Conclusions:**

A dyad‐centric approach to analysis was useful for observing how parents and adolescents engaged in resistance together. Our findings suggest that resistance can be seen as a dyadic concept that reflects a personalized relationship history that has implications for parent and youth identity development.

The purpose of this paper was to identify and describe dyadic resistance in parent‐adolescent interactions in a sample of Canadian parent‐adolescent dyads. Resistance was defined as an expression of agency to counter another's attempted influence or control (Brehm 1966; Brehm and Brehm 1981; Goffman 1961; de Pano 2023; Raby 2005). Researchers of child and adolescent development have defined resistance to parents' directives and requests as legitimate and important agentic responses (Kuczynski, Burke, and Song‐Choi 2021; Van Petegem et al. 2015; Vansteenkiste et al. 2014) signaling normal and healthy manifestations of developing autonomy (Crockenberg and Litman 1990; Kuczynski and Kochanska 1990; Kuczynski et al. 1987). This developmental definition of resistance attends solely to children's or adolescents' behavior. Such a view of adolescents' resistance to parents is limited by the emphasis on one side of the relationship even though there has been significant research on parents' resistance of their children (Marshall et al. 2014; Flamant et al. 2022; Leung 2021; Young et al. 2005; Tilton‐Weaver and Marshall 2017). Face‐to‐face resistance, or resistance that is co‐constructed and involves the expression and navigation of differences during parent‐adolescent interactions within the context of a longstanding relationship, has received less research attention. To attend to this shortcoming, this study adopted a dyadic level of analysis, examining how pairs of adolescents and parents resist one another during face‐to‐face interactions.

## 1 Research on Expressed Resistance

Previous studies on expressions of resistance have examined how children from various age groups from toddlerhood to adolescence expressed their agency in response to parental limitations and how parents perceived and experienced such expressions (Burke and Kuczynski 2018; Kuczynski, Burke, and Song‐Choi 2021; Kuczynski and Kochanska 1990; Parkin and Kuczynski 2012; Robson and Kuczynski 2018). These studies have described a variety of interpersonal influence strategies ranging on dimensions of overtness, cooperativeness (Parkin and Kuczynski 2012), and skill (Kuczynski, Burke, and Song‐Choi 2021). Parents described their perceptions of their children's resistance in terms of social competence, assertiveness, and annoyance (Kuczynski, Burke, and Song‐Choi 2021). Their responses to such resistance were described in terms of power‐assertive strategies like punishment and threats to autonomy supporting strategies like accommodation and reasoning (Burke and Kuczynski 2018). Taken together, this body of research has described how children and adolescents resisted their parents attempts to control, guide, or influence them, and how their parents interpreted and responded to their children's resistance. Findings from these studies on resistance have tended to focus on how children resist their parents; however, researchers who study adolescents have also addressed parental resistance.

Researchers who study adolescents have expanded on what is known about resistance by moving beyond asking about or observing children's strategies to reveal how parents resist their children in everyday conversations. Evidence suggests that parents resist their children's attempts to assume authority over their activities (Marshall et al. 2014) or regulation expectations (Young et al. 2008). Disputes between adolescents and parents have been found to reflect parental concerns about where the boundaries should be between adolescents' personal jurisdiction and the legitimate authority of their mothers (Seyed Mohammad Assadi et al. 2011). Mothers were found to be more willing to give their children options, compromise, and negotiate more when conflicts arose over disagreements about personal choices in service of the formation of a child's sense of agency and self (Nucci and Weber 1995). On the other hand, mothers have been found to establish boundaries, set limits, and exercise their authority when their children's actions contravened social conventions or entailed actions that posed a risk to their children or others (Nucci and Smetana 1996). Parents will negotiate more and give choices to their children over personal issues like their clothing and music choices, friends, and recreational activities based on the desire to support their identity and self‐expression (Nucci and Smetana 1996). Parents will, however, resist their children's attempts for autonomy on matters where they feel their authority is legitimate (Nucci and Weber 1995). Such studies have expanded understandings of parental resistance. They have also elucidated how parent‐adolescent conflict and resistance relate to the boundary between personal and prudential or conventional issues (Smetana 1995; 2005).

Research on information management has helped to fill in gaps on what is known about resistance by addressing how adolescents express their agency when communicating with their parents about their activities and whereabouts (Marshall, Tilton‐Weaver, and Bosdet 2005). Most adolescents engage in withholding information from their parents (Darling et al. 2006), and a wide range of strategies has been identified and reported that adolescents use strategically to withhold information (Darling et al. 2009). Adolescents have been found to leave out information, avoid, use partial disclosure, acts of omission, and deception to withhold information from their parents (Cumsille, Darling, and Martínez 2010). Strategies preferred by adolescents are those that are less evasive like waiting for parents to ask, or omitting details, over more evasive strategies like lying and keeping secrets (Laird and Marrero 2010). The decision to withhold information by the adolescent can depend on whether activities fall under the jurisdiction of the parent or adolescent, whether disclosing will maintain family‐related activities or secure social support (Marshall, Tilton‐Weaver, and Bosdet 2005). Adolescents have been found to be motivated to disclose out of feelings of obligation to obey, and non‐disclose out of fear of negative emotional repercussions or punishments (Darling et al. 2006). The information management literature has been useful for highlighting the active role children play in their own socialization (Maccoby and Martin 1983) as well as how the strategies young people use can depend on adolescents' anticipated responses from their parents.

The quality of the parent‐adolescent relationship plays an important role in what adolescents choose to keep private (Smetana et al. 2006). Smetana et al. (2010) found that adolescents' secrecy with parents can be characterized in terms of daily changes and individual differences in the parent‐adolescent relationship's quality and problem behavior. Tilton‐Weaver and Marshall (2017) reported that from the perspective of some adolescents, managing the information they provide to their parents supports establishing privacy and autonomy boundaries, while allowing them to preserve important relationships. The literature on information management has aided in broadening understanding of how parents and adolescents engage in resisting one another by elucidating the strategies adolescents use to preserve boundaries without harming their relationships with their parents.

Previous research on the various ways parents and adolescents resist one another has used methods focused on the individual level of analysis. However, studies conducted at the individual level may overlook in their analysis the bidirectional nature of parent‐child relationships (Bell 1979; Lollis 2003; Loulis and Kuczynski 1997). Focusing on the individual de‐contextualizes behavior by overlooking the adolescent‐parent relationship in which resistance occurs and may do little to aid in reframing resistance as developmentally appropriate assertions of agency (Ginsburg and Rodriguez 2020). As such, research is needed that examines in depth how resistance is given shape and meaning by the shared unique parent‐adolescent relationship context. Since the focus in the literature on adolescent‐parent resistance has tended to be on parent or adolescent strategies, it is not yet known what resistance looks like face‐to‐face during parent‐adolescent interactions. Finally, the way parent‐adolescent disputes unfold is dependent on the uniqueness of each dyad (Hinde 1987; Lollis 2003). It is, therefore, important to acknowledge that resistance strategies are bound and shaped by multiple contextual influences. For this exploratory study we were curious about whether resistance can be observed as a dyadic process. We used the concept of face‐to‐face resistance to expand on the resistance literature by describing patterns that were involved when adolescents and their parents engaged in expressing and navigating differences during their interactions.

One way of studying face‐to‐face resistance patterns in interactions was to observe natural points of potential differences between parents and children. The transition to high school represents a period of renegotiation across multiple domains in the lives of young people and their families (Benner 2011). It is a planned and anticipated family transition wherein young people learn to manage new academic demands and balance family, leisure, and extracurricular activities (Marshall et al. 2014). Most parents are involved in the transition to high school (Falbo, Lein, and Amador 2001) and provide support and stability throughout (Benner 2011). The high school transition occurs in early adolescence, a period known for increased parent‐adolescent conflict (Weymouth et al. 2016). The transition to high school was examined because it is an opportune moment to observe the multiple ways parents and adolescents jointly raise, navigate, and conclude discussion on topics about which expectations, roles, and responsibilities may need to be recalibrated. As such, we chose to observe a group of parent‐adolescent dyads talking about the upcoming transition to high school to observe how families engage in face‐to‐face resistance together.

## 2 Theoretical Considerations

Many theoretical concepts in the social sciences, despite being intrinsically interpersonal, have been studied by examining individuals in isolation. As in other close relationships, interactions in parent‐adolescent dyads create non independence due to the shared history that overlays and influences each shared interaction (Cook 2001; Lollis 2003). Such interactions occur within the context of a longstanding relationship (Hinde 1976). Influence in parent‐child interactions has been found to be reciprocal and driven by relationship‐specific factors (Cook 2001). The history of dyadic interactions that are the building blocks of the relationship feeds into current and future expectations that parents and children can have for one another (Hinde 1987). These histories of interdependent interactions make each relationship unique and irreducible. Relationships cannot be reduced to individual characteristics of parents and adolescents, and each relationship is distinctive with characteristic patterns of engaging in sequences of interactions together (Marshall et al. 2023; Saxbe, Rodriguez, and Margolin 2013). Thus, the parent‐adolescent relationship is comprised of past face‐to‐face interactions and experiences that are likely to shape how members make sense of interactions in the present. This way of sense‐making is likely to be an integral part of how dyad members engage in interactions together. To understand how such interactions might be face‐to‐face resistance, we briefly present an overview of family conflict theory to note the push and pull of two opposing forces in parent‐adolescent relationships.

Sprey (1979) proposed that a perpetual tension exists within every social group, especially small and intimate ones like families, between the joint pulls of autonomy and togetherness. Parents and adolescents navigate their differences together in the small moments to avoid escalating tensions to damaging levels or reducing them to separate individuals (White, Martin, and Adamsons 2019). As such, conflict as a dialectical process can play an adaptive role in helping families achieve greater unity (Simmel 1904). We did not study conflict in the present study but drew from family conflict theory to highlight the importance of understanding how families engage in navigating the push and pull of autonomy and togetherness during their interactions.

Even though the joint pulls of autonomy and togetherness may be present in both family conflict and face‐to‐face resistance, adolescent expressions of agency have sometimes been shown to not involve direct confrontation inherent to family conflict (Parkin and Kuczynski 2012). Parents and adolescents may find ways to sidestep, slip, or otherwise avoid direct confrontations with each other. Expressions of resistance in parent‐adolescent interactions are believed to be adaptive because they can signal a need to renegotiate family rules (Soenens and Vansteenkiste 2020); however, studies that systematically examine the specific processes by which parents and adolescents engage in face‐to‐face resistance are limited. As such, this study helped fill this gap in our knowledge. We proposed that face‐to‐face resistance be conceptualized as a dyadic concept involving the expression and navigation of different viewpoints from both partners. Conceptualizing face‐to‐face resistance as a dyadic concept allowed us to describe both parental resistance as well as the patterns that parents and adolescents engage in when they are resisting one another. We suggested that describing patterns of face‐to‐face resistance in parent‐adolescent interactions may contribute to our understanding of how the parent‐adolescent relationship evolves during adolescence.

### 2.1 A Social Constructionist Approach

We chose to approach the data used in this investigation from a social constructionist perspective because of how we see family interactions. According to Hinde (1976), relationships are created by two people interacting over time. Consequently, each parent‐child interaction is simultaneously influenced by a history of mutual interactions as well as prospects of future interactions (Lollis 2003). Social constructionism is rooted in the view that “what we take to be the truth about the world importantly depends on the social relationships of which we are a part.” (Gergen 2015, p. 3). Social constructionist inquiry is concerned with how knowledge is socially produced and reproduced through communication by people in interaction (Berger and Luckman 1991). Interactions, in this sense, are the stuff and substance of interpersonal relationships (Berscheid 1995). The presumption that personal experiences like parent‐adolescent interactions shape how people make sense of their worlds is aligned with a relativist ontological position. A relativist ontological position challenges the idea that a singular reality exists independent of human practices and sees reality as contingent, local, and multiple (Braun and Clarke 2022). We applied the broader process of social construction and meaning making in relationships as a lens from which to view face‐to‐face resistance as co‐constructed during interactions and informing, as well as being informed by, the parent‐adolescent relationship. This point of view acknowledges that interactions are bilateral and occurring in a relational context. The co‐construction of meaning by parents and adolescents inherent to social constructionism aligned well with the objective of this study to describe face‐to‐face resistance during interactions.

The goal of social constructionist inquiry is not to uncover a definitive final word about a phenomenon but instead seek to offer a partial “reading” of the data of how face‐to‐face resistance can look to show how participants make sense of their reality and bring these realities into being. We conceptualized face‐to‐face resistance as socially constructed by parents and adolescents in interaction within the enduring context of their unique relationship. We sought to identify features of face‐to‐face resistance that were unfolding in the context of a relationship that, while not visible, have been and continue to play out over years.

### 2.2 The Present Study

Understanding the social meaning of face‐to‐face resistance requires an understanding of the context in which it occurs; therefore, we asked the following question: What are the co‐constructed patterns of face‐to‐face resistance that parents and adolescents engage in during conversations about the upcoming transition to high school? We expected that face‐to‐face resistance can be observed during interactions as a dyadic process, and that parents and adolescents construct resistance together in ways that reflect the autonomy‐togetherness tension inherent to close relationships.

## 3 Material and Method

### 3.1 Participants

Data were collected from 27 parent‐adolescent dyads recruited from two major Canadian urban centers where youth attend high school after elementary school without attending middle school. Twenty‐eight dyads initially expressed an interest in participating but one of the dyads did not end up participating. Data from 26 of the remaining 27 dyads was reported in a longitudinal study of the transition to high school (one dyad dropped out after completing the first interview; Marshall et al. 2014). Analysis of data was conducted on the 27 dyads who completed the first interview of the longitudinal study. The two cities have different pathways to high school. In one city (*n* = 15 dyads), students can choose which high school they attend for grades eight to 12. In the second city (*n* = 12 dyads), students from feeder schools move to area high schools for grades nine to 12. As such, the range of ages for adolescents who planned to go to high school the following year varied. Twelve of the remaining adolescent participants were 12 years old, nine were 13 years old and six were 14 years old (Median = 13.27). Fifteen youth identified as female and 12 identified as male. Four of the youth were born outside of Canada but had lived in Canada from anywhere between one to 11 years (Median = 3). Three adolescents were receiving academic supports at school.

Only one parent was invited to participate despite most adolescents having contact with both of their parents. Including both parents would have added more complexity and cost to data collection and analysis. The age of the participating parents ranged from 35 to 50 (Median = 44 years). Twenty‐four parents were mothers and three were fathers. One participant was a step‐parent and nine parents were single. Most parents were employed (*n* = 22). The educational background of parents ranged from completion of high school (*n* = 6) to college diploma (*n* = 7) to undergraduate (*n* = 10) to graduate degree (*n* = 4). In terms of ethnic background, the majority (*n* = 20) were of European descent and none were French speaking families, five dyads were Asian, and two dyads reported other backgrounds. Nine parents were not born in Canada with years of residency in Canada ranging from one to 40 (Mean = 15.4). Four dyads reported speaking a language other than, or in addition to, English at home (i.e., Nepalese, Mandarin, Cantonese, and Spanish).

### 3.2 Procedure

Data analyzed for this study were originally collected between 2010 and 2012 to examine the joint projects of parents and adolescents during the transition to high school using the Action‐Project Method (A‐PM) (Young, Valach, and Domene 2005). However, for this study, a different type of analysis was used to describe face‐to‐face resistance processes specifically. Collection of original data followed ethical review by the Behavioral Research Ethics Boards of the two universities at which the two Principal Investigators were employed. Participants were recruited through advertisements distributed to students in the spring (May and June) of their final year of elementary school. All students enrolled in elementary schools in the smaller city received advertisements and eight elementary schools were selected at random for distribution of advertisements. In addition, advertisements were placed in newspapers and online, as well as at leisure activity centers to recruit dyads in the early summer months (June and July). Initial screening interviews were conducted with parents to verify that the adolescents were starting high school the following September and had not been diagnosed with a serious cognitive impairment or mental illness. A separate phone call with adolescents was made to ensure their interest in participation. Inclusion criteria were that both the parent and adolescent agreed to participate and both partners could communicate in English. Participants completed a self‐assessment in that they were asked their opinion about whether they thought that they could communicate effectively in English. Participants' transportation expenses were reimbursed because interviews were conducted at the university. In addition, each dyad member was paid an honourarium of $40.00 (CDN) for their participation.

The decision to use an existing data set was founded upon cost‐efficiency, low risk to participants (Langkamp, Barnes, and Zuckerman 2021), and avoiding undue burden on individuals (Doolan and Froelicher 2009). The data set allowed for observing microprocesses that occur during moment‐to‐moment partner interactions (Gottman and Notarius 2000), were multi‐informant providing perspectives from both parent and adolescent (Bernard 1982; De Los Reyes, Ohannessian, and Racz 2019), and multi‐source with video recall interviews providing information on how individuals make meaning of resistance (Welsh and Dickson 2005). A dyad‐centric analysis using this observational data was conducted to address a gap in what is known by examining how face‐to‐face resistance unfolds in this sample of non‐clinical parent‐adolescent dyads. The data collection in the original study involved a three‐part conversation in private research rooms at universities at two Canadian urban centers. All interviews followed the same three‐part format using the same script.

1. Warm‐up Interview
  Part one was an unstructured video‐recorded introduction and warm‐up interview. Two trained research interviewers explored the dyad's views about the upcoming transition to high school and what they were doing together currently regarding the transition. The interviewers started this warm‐up by asking the adolescents about what they did in their leisure time and how they organized these activities, as well as what they anticipated during the upcoming transition to high school. Parents were asked how they were involved in adolescents' leisure activities and high school preparations. The two interviewers asked questions of both adolescents and their parents, but the questions were open‐ended to allow the parents and adolescents to steer the conversation. The interviewers asked the dyad near the end of this warm‐up to select a topic of their choice from the warm‐up interview to have a conversation without the interviewers in the room. The purpose of the warm‐up interview was to ensure parents and adolescents felt comfortable in the research setting, to orient dyads to conversing on a topic related to the transition to high school, and to ensure that both parent and adolescent were included so that neither the parent or adolescent alone would drive the conversation.
2. Self‐directed Conversation
  Participants were instructed to have a conversation about the topic they had selected during the warm‐up interview and allow the conversation to evolve until it came to a natural end at which time they could open the door and invite the interviewers back into the room. Once the dyad selected a topic, they were instructed to converse in private without the interviewers present and advised that their conversation would be audio‐ and video‐recorded. They were instructed to converse until they felt the conversation had concluded, at which point they were to directed to knock at the door to invite the interviewers back into the room. The self‐generated and self‐directed video‐recorded conversation without interviewers present took place immediately after the warm‐up interview.
3. Video Recall Interview
  In part three, each participant went to a separate room with one of the interviewers to view a playback of the self‐directed conversation. Participants were asked during the video recall interview to report on their thoughts and feelings during sections of the self‐directed conversation at approximately 1‐min intervals. That is, the interviewer instructed the participant that the playback would be paused at 1‐min intervals, and that the participant would be asked at each pause to respond to the following three questions: (1) During that segment, what were you thinking? (2) During that segment, what were you feeling? (3) Do you have anything else to add about the segment you just watched? The same three questions were repeated for each 1‐min interval for the entirety of the self‐directed conversation.

The audio recordings of the self‐directed conversations and video‐recall interviews were professionally transcribed verbatim. In addition to transcripts, other components of communication were noted and described (i.e., laughter, pauses, or variations in inflections or tone of voice). Self‐directed conversations averaged 10:24 min in length; video recall length averaged 27:29 for adolescents and 35:24 for parents. Participant confidentiality was ensured by assigning each dyad an identification number and removing names or any other identifying information from interview transcripts. Transcribed interview data as well as information linking participants with transcriptions were stored in two separate locked filing cabinets in secure labs at each respective institution where the data were collected. Parents and adolescents did not see each other's transcripts for the video recall. Participants were told the other person in their dyad would not view their responses to the video recall interview, which may have enhanced the likelihood of parents and adolescents being forthcoming during the video recall interviews.

### 3.3 Analysis

**Figure 1:**
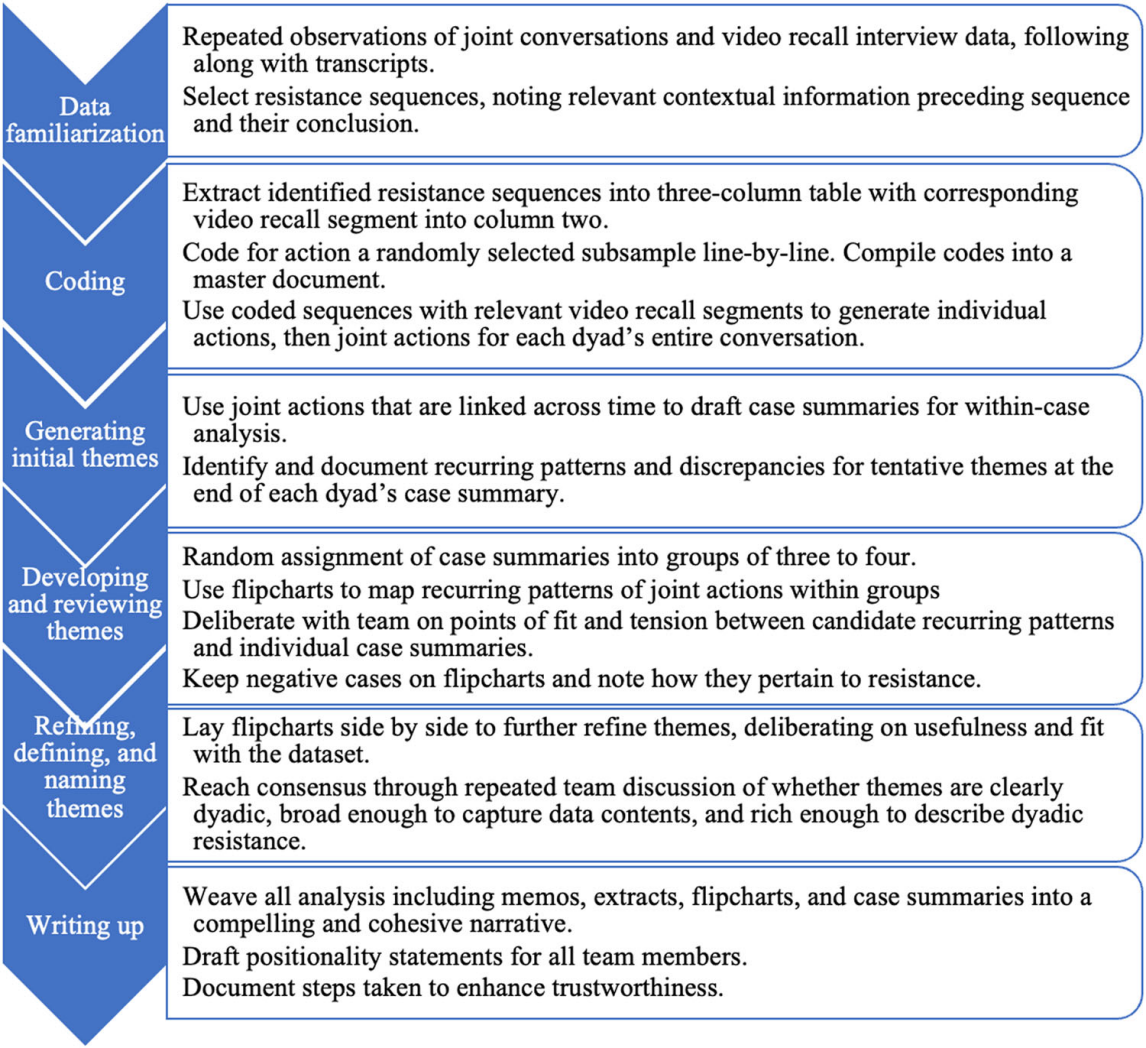
Visualization of six phases of analysis.

A critical reflexive thematic analysis guided by a relativist ontology and social constructionist theory was conducted on the data (Berger and Luckman 1991; Braun and Clarke 2022; Charmaz 2017). Critical reflexive thematic analysis is suitable for capturing semantic and latent meanings around a topic, offering descriptive and interpretive accounts of data (Braun and Clarke 2022). In line with a social constructionist theoretical framework, critical reflexive thematic analysis foregrounds that interactions shape and are shaped by a unique and shared parent‐adolescent relationship. The theoretical flexibility of thematic analysis means it can be informed by a social constructionist approach concerned with conceptualizing face‐to‐face resistance as more than adolescent behavior to see how parents as well as adolescents resist one another. As such, critical reflexive thematic analysis is appropriate for examining in depth what patterned meaning individuals make when they resist one another within a longstanding relationship context. Braun and Clarke's (2022) six phases provided the initial guiding framework for describing our approach to organizing and analyzing the data. These six phases are: (1) data familiarization, (2) coding, (3) generating initial themes, (4) developing and reviewing themes, (5) refining, defining, and naming themes, and (6) writing up (Braun and Clarke 2022). However, the phases presented here deviate in that we devised an approach for developing themes of face‐to‐face resistance at the dyadic level of analysis. The six phases are summarized visually in Figure 1.

1. Data Familiarization
  Regarding familiarization with the data, the first author had been observing the video conversation and video recall data for over 5 years, developing tentative understandings of face‐to‐face resistance. The second author who supervised analysis was one of the principal investigators responsible for the collection of the initial data and was therefore, already highly familiar with the data. The third author provided feedback on drafts of the manuscript but was not engaged in coding or analysis. As such, they did not watch or listen to the recordings. After repeated reviewing of the videos and transcripts, the authors met to discuss how to identify and describe face‐to‐face resistance in the data that varied in length, were goal‐directed, and embedded in conversations. Resistance sequences were selected as our unit of analysis given our goal of describing how dyads expressed and navigated their differences together. A resistance sequence begins with an expressed difference by a dyad member from the other's perception, opinion, or attempt to control them. We used a combination of the first author's theoretical and empirical knowledge about resistance in combination with reflections from video recall interviews to confirm the speaker's intent to resist from the corresponding section of the self‐directed conversation transcript. We captured context for each sequence by noting relevant preceding information for each sequence (i.e., topic discussed, how it was raised, and how members differed). The entire exchange following the initial difference was included and the conclusion of each sequence was identified as the point at which no further back and forth occurred between parents and adolescents on the topic being discussed.
2. Coding
  Once a unit of analysis was selected, coding of the data was initiated (Braun and Clarke 2022). The research team that conducted the analysis consisted of the three authors. The first author coded all the transcripts. The second author provided ongoing supervision in the form of team discussions about the coding and theme development. The third author provided feedback on the codes and themes. To aid the coding process, we recruited a researcher in social work as well as a clinical psychologist, both with extensive experience observing children and families to help conduct independent checks of our codebook. The first author extracted resistance sequences into a separate document in a single column (column one) along with sections from each member's recall interview that corresponded with the resistance sequence in a separate adjacent column two (see Table 2). This was done to clarify intent to resist and obtain a deeper understanding of each member's account via their shared thoughts and feelings during each sequence. Ambiguous cases were discussed between the first and second author until agreement was reached. The first author began step two by coding a randomly selected sub‐sample of extracted sequences from three cases. The first author assigned code names line‐by‐line with a key phrase thought to signify each person's action. Interpretation during coding was guided by the research team's theoretical perspective, the first author's understanding of the resistance literature in conjunction with recall interview information.

Tentative codes were compiled into a codebook including the name, definition, and a verbatim example. Table 1 shows examples of codes with definitions and an example. A researcher with 17 years of experience observing children and families then independently coded three additional cases with the first author using the codebook that was developed from the first three cases. The researcher's feedback was tracked through memos and changes were incorporated via discussion between the researcher and the first author. Disagreements about sequences were resolved through discussion between the first author and the researcher. The content of the discussions about coding disagreements was discussed further during meetings with the research team and was used to inform revisions to the initial codebook. Once the codebook was revised, the remaining cases were coded by the first author with ongoing journaling of what dyads appeared to be doing together throughout. The authors met once most cases were coded to discuss journal entries and how to capture phenomena at the dyadic level of analysis. Finally, a clinical psychologist with experience observing families with children independently coded a final case using the revised codebook; this time little difficulty was reported in applying the codebook. No disagreements were observed between coders.

**Table 1:** Examples of codes with definitions and exemplars.

Code name	Definition	Exemplar
Deferred response or agreement	Intentionally not responding or stalling to respond to other's request or plan. The goal may be to hold out for a better offer, signal reluctance to acquiesce, or avoid being drawn into further conversation on a potentially contentious topic where concessions might be requested. Sometimes, not responding can be an expression of dissatisfaction, resistance to suggested change, or even anger in a way that will not elicit reprisal. Look for words like “Um…,” “it depends,” or “we'll see.”	P: OK um well do you have anything else to add to this conversation? A: N‐mm no… mm… mm. No… not really.
Slip it in	To reveal potentially important and or unexpected information in passing and with limited details. The goal is to test the waters around anticipated consequences or punishment from telling, permission, disappointment, or potential confrontation about a topic by providing as little information as possible.	P: No, I'm, I'm good with it. I mean, I see that you're taking responsibility for your bad marks in math. A: And geography. P: What? A: Never mind, hee hee.
Abrupt topic change	A sudden or abrupt change in topic which can occur when tensions are mounting on a topic. Goal is to shift other's attention by steering the conversation to a less contentious topic (“Anyways…,” “Um, another thing is…”) or to return to a topic of ongoing concern that was raised earlier in the conversation	P: That's, that's one of the things that I'm concerned about, is you not being able to say “No I've had so much today already.” A: No I, I probably have. P: OK you saucy little thing. Um, and I think another big thing is/

Next, we devised an approach to analyzing dyadic conversation data. A third column was added to the coding document consisting of three rows. The first row described the youth's action during the sequence based on a combination of the first author's interpretation of the coded extract and the concomitant information from their recall interview. The second row contained a description of the parent's action using the same process as for the youth. The third row described the joint action in which both individual actions were brought together to describe what the dyad members appeared to be doing together. This three‐step process was applied to all sequences, an example is provided in Table 2.

**Table 2:** Generating joint actions using coded data with video self‐confrontation.

Parent: So where do you wanna start [daughter's name]? Adolescent: I don't care ‐ Minimal response Parent: You don't care? Do you wanna go for the gusto on the big issue first? Or do you want to pussy foot around some little issues? ‐ Multiple successive questions ‐ Restrict permissions or offer a limited range of choices Adolescent: Pussy foot ‐ Minimal response Parent: Pussy foot. OK how d'you think you, how d'you think high school's gonna change our relationship? ‐ Capitulate or relent	Adolescent self‐confrontation: Adolescent: and she thinks like her way is the right way about everything Adolescent: No. It was just kinda like adrenalin like, I hope this is over soon Researcher: Just ‘cos OK so your mum asked you do you wanna start with the big issue? And what could that be? Adolescent: I dunno Researcher: You don't know? Adolescent: No Parent self‐confrontation: Parent: Well, I know that [daughter's name] eh‐uh‐feels that we're too strict with her: that we never let her do anything, that/ Researcher:/Right Parent: that's her big issue Researcher: OK fine Parent: and that is her big complaint is that [words stretched out in emphasis] “I never get to do anything” Researcher: OK so you were thinking that all this/stuff's going on/ Parent:/Let us go for it/because I know that that is her major thing	Adolescent: provide minimal responses to avoid discussion about what appears to be a major issue.
		Adult: Attempt to discuss ongoing major complaint of youth that mother is too strict by giving adolescent options that include the ‘big issue.’
		Joint: “Pussy foot” around discussing a sensitive topic

*Note:* The left column shows the coded excerpt. The middle column shows the corresponding segment from each dyad members self‐confrontation interview. The right column shows the action of both parent and adolescent, and the joint action that was developed based on the individual actions, respective self‐confrontation segments, and coded text.

Table 2 shows an example of how joint actions were generated. The codes in column one represent what the youth and parent appeared to be doing at the individual level. The corresponding video recall excerpts in column two were used to examine the parent's and adolescent's thoughts and feelings about their actions during the resistance sequence and was used to gain clarity about the intention that the individual assigned to the coded actions in column one. The third column shows our attempt to bring the goal‐directed actions of parents and adolescents together in a way that allowed us to capture the complexity of interactions and describe how each partner's actions created interdependence. Put another way, column one represents coding of individual elements of resistance, column two represents how the individual actions were directed towards a goal, and column three describes how the goal‐directed actions of two individuals come together to be co‐constructed face‐to‐face resistance. Examples of joint actions using the same processes included debating the fairness of household rules or expectations, comparing the relevance of past situations or actions to present ones, and clarifying perceptions of past events and the parent's or adolescent's role in them. The method of generating joint actions developed for this study is unique to thematic analysis approaches in that we brought together qualitative data from two sources (conversation transcripts and video recall interviews) and two informants (parent and adolescent), allowing us to move to the dyadic level of analysis as well as potentially enhancing the credibility of our findings (Tracy 2010).

3. Generating Initial Themes
  The joint actions were ordered in sequence from start to finish for each dyad's conversation in the form of a case summary. These summaries provided an overview or snapshot of each dyad's face‐to‐face resistance from initiation to duration to conclusion. Ordering codes sequentially via case summaries provided a basis from which we could begin to document how back‐and‐forth patterns of face‐to‐face resistance unfolded during interactions. Within case analysis through writing case summaries represented a first step towards abstraction from coding to the linking together of joint actions. At the same time, conducting within‐case analysis in the form of a case summary helped to ensure that analysis remained close to the data and facilitated the location of where the ideas for themes originated from in the data set. We drew from the case summaries to generate ideas for initial themes, which mapped onto phase three of our thematic analysis (Braun and Clarke 2022). We documented initial themes at the end of each case summary. This within‐case analysis also facilitated examining whether there were similarities or recurring patterns between dyads in the next phase of analysis.
4. Developing and Reviewing Themes
  Once all 27 case summaries were written, we moved to the fourth phase of developing and reviewing the themes we had been generating through conducting a cross‐case analysis of the case summaries (Braun and Clarke 2022). Dyads were assigned through random selection without replacement (i.e., case IDs in a hat) into groups four cases at a time (for one group there were three cases because the total number of cases was 27). The total number of groups was seven. Summaries were then read repeatedly while making notes on flipchart paper to map recurring patterns of joint action within groups. Examples of recurring patterns include “sharing versus extracting information,” “relationship maintenance strategies,” “messing with,” and “conversation steering.” Any recurrences we mapped on the flipcharts were discussed by the team with respect to the ideas for initial themes that were generated in the case summaries from step three. In other words, it was the candidate themes from step three's case summaries that served as the springboard for discussion among the team about which potential patterns may transcend dyads and which were unique to a dyad.
  Individual case summaries were compared repeatedly against candidate patterns of face‐to‐face resistance during research team discussions to facilitate movement of resistance to a higher level of abstraction. Hence, a case analysis framework was used within a thematic analysis methodology to refine the developed patterns from the cross‐case analysis. Four groups included dyads where no face‐to‐face resistance was observed. These cases remained part of the analysis to compare against dyads where sequences present to achieve a richer understanding of face‐to‐face resistance in a section we named “negative case lessons.”
5. Refining, Defining, and Naming Themes
  For phase five, the flipcharts were then laid side‐by‐side to define, name and refine themes were deliberated on with respect to their names, usefulness, and fit with the data by the authors (Braun and Clarke 2022). Discussion centered on whether themes were clearly dyadic, broad enough to capture data contents, and rich enough to describe face‐to‐face resistance. Once the themes were agreed upon by all authors, all journal entries, memos, summaries and maps were woven together into a report of how data were used to address the research question (Braun and Clarke 2022). Memos and journals were used to track the analytical process as well as to draft a reflexivity statement to describe how we engaged in construction as instruments throughout analysis.
6. Writing Up
  Phase six (writing up) consisted of bringing together all analysis, memos, extracts to form a compelling narrative about the topic (Braun and Clarke 2022). We describe how memoing, trustworthiness, and positionality were woven throughout our analytical process before moving to results. Throughout analysis, we found it useful to interrogate emotional reactions to the video conversational data (Braun and Clarke 2022). Through journaling and discussion, we interrogated the clinical urge to problem‐solve, realizing that seeing interactions in conflict terms came with observer‐centric assumptions and presuppositions about how people engage. Conversely, maintaining a focus on actions through ongoing memoing created a more open and empathetic stance that helped us refrain from jumping to conclusions about dyad members. Moreover, focusing on actions aided us in seeing behaviors in context, emphasizing what individual dyad members were trying to do with their actions.

Trustworthiness in this study was enhanced using video recall interviews as a form of triangulation and member‐checking (Guba and Lincoln 1989). Participants clarified during recall their intent to resist as well as the goals of their verbalizations or behaviors during sequences. Another form of member checking was to check in regularly with the second author who was the person who had overseen data collection. Video recall interviews assisted with triangulation to support confirmability and authenticity in interpretation. Prolonged engagement with the data over an extended period in the form of persistent and ongoing observation of the video conversations and transcripts assisted with immersion to enhance researcher credibility. As mentioned, a reflexive journal and memos were maintained throughout analysis, which were used to write a reflexivity statement describing challenges faced to support credibility as well as articulating the theoretical lens guiding the analysis as constructing researcher to describe positionality. Moreover, records of conversations with the co‐authors and other researchers were maintained in a running document of decisions associated with how data were analyzed to connect decisions to findings. These records act as an audit trail to support credibility (Cope 2014). Reporting standards follow those recommended by the American Psychological Association for qualitative inquiry (Levitt et al. 2018).

### 3.4 Positionality Statement

The interviewers who collected the original data were graduate students in social work, education, and family relations and human development. These students were chosen deliberately for their professional skills and experience they brought to interviewing families in a research context.

The first author of this paper has 8 years of child protection experience with families with adolescents. During this time, he has observed and supported parent‐adolescent dyads experiencing conflict. He also has had experiences working in partnership with contracted parent‐teen mediators and witnessed the variety of approaches they brought to managing parent‐adolescent conflict. The first author engaged in ongoing reflection on the way their professional stance as a social worker may have emphasized problem solving. Ongoing checking with team members was helpful in protecting against viewing the conversations in terms of the presence or absence of conflict, which dyad member was the source of the conflict, and the degree of conflict (high or low).

The second author of this paper specializes in research on psychosocial development in adolescence with an emphasis on contexts of peer and family relationships. She has researched parent‐adolescent interactions using both qualitative and quantitative approaches to data collection and analysis. Her lived experience parenting foster children during their adolescence contributes to her worldviews of family dynamics such as parent‐adolescent resistance.

The third author has practice and research experience in the fields of child and youth care and child welfare. His research emphasizes children's rights and improving interdisciplinary services to youth.

## 4 Results

In this section we present our plan of analysis for the findings. To begin, descriptive statistics were used to provide an overview of what face‐to‐face resistance looked like in the conversations. Before presenting the themes that we developed, we summarize the number of resistance sequences that we counted, prevalence as well as who expressed difference first, the number of sequences per conversation and timing of first sequence, as well as topics that dyads differed on. We then present our themes which we developed following the analytical steps described in the previous section.

In total, 97 resistance sequences met unit of analysis criteria of one member expressing a difference from their partner's perception, opinion, or attempt to control them, occurring at least once in 23 of the 27 conversations (85.19%). Adolescents (*n* = 57, 58.16%) expressed an initial difference more often than parents (*n* = 41, 41.84%). Dyads varied on how many sequences of face‐to‐face resistance were in their conversations (range = 0−9, Mean = 3.63, SD = 2.69). The time at which first resistance sequences were observed in conversations varied from dyad to dyad, with sequences starting as soon as the researchers left the room in 12 of the self‐directed conversations (range = 0−18:50, Mean = 2.28 min, SD = 4.72 min).

Consistent with previous research on parent‐adolescent conflict, dyads in this sample generally differed on topics related to matters of everyday life (Montemayor 1983). These included expectations about responsibilities like schoolwork and extracurricular activities, communication, unstructured time, substance use and dating. Also consistent with the literature on parent‐adolescent conflict that it is low‐level and commonplace for many families (Weymouth et al. 2016), face‐to‐face resistance was usually tentative and rarely escalated to full‐blown, prolonged confrontations. We suggest that face‐to‐face resistance may be similar in that we observed it to be low level and related to mundane family matters.

We developed four distinct themes to describe how the parent‐adolescent dyads in this sample navigated differences together as well as capture a pattern of tension associated with each theme, presented below with illustrative examples from the data. In descending order of frequency, the themes were *Reminding of and then defending the “constant battle” lines* (*n* = 33), followed by *Cautious avoidance* (*n* = 30), *the Nudging match* (*n* = 24), and *No point anymore and minimal responses* (*n* = 10). Table 3 offers a summary of the four themes developed with their operational definitions and an exemplary quote for each.

**Table 3:** Summary of face‐to‐face resistance themes.

Theme name	Operational definition	Exemplary quote
Reminding of and then defending the “constant battle” lines	Characterized by boundaries being re‐drawn and defended. Dyad members take a hard‐line approach to face‐to‐face resistance by cajoling, over‐talking, and drawing bottom lines. Tension: Slap‐shotting and blocking with both partners simultaneously taking slaps and blocking each other's shots.	P: You're not writing stories mm, if you don't like it, you'll just dislike it more and more. You just need to get used to writing more. A: Okay. P: And you can be better … A: … [noises of fidgeting] P: (says shortened version of youth's name to get his attention)? A: What? P: You have to do work… A: Sometimes
Cautious avoidance	The dyad treads cautiously around a sensitive topic (i.e., difficult, upsetting, or cause of concern for one member) that is potentially contentious and possibly discussed in the past. Tension: Avoiding (“Do I get to go home now?”) or approaching (“And the big thing for me is…”) a topic.	P: So where do you wanna start? A: I don't care. P: You don't care? Do you wanna go for the gusto on the big issue first? Or do you want to pussy foot around some little issues? A: Pussy foot. P: Pussy foot. OK how d'you think you, how d'you think high school's gonna change our relationship? A: [whispers] Our relationship? Um… I think I will get away more. P: ‘Cause you want more freedom.
The Nudging match	Characterized by perception disputes over a topic's importance and whether continued discussion about it is needed. Tension: Magnifying and shrinking. Magnification involves calling the other's attention to a topic's importance by pressing them to contemplate it in detail (e.g., using hypothetical situations to encourage planning for contingencies). Shrinking involves downplaying a topic's significance (e.g., “She doesn't have to be worried,” “calm down”) or distracting.	P: Yeah like if I don't know exactly where you are and when I ask you just sort of say you y'know [funny voice] “I don't know we're just gonna hang out” [slight laugh]. A: But I don't know so that's why I say it. P: I know and that's true, you don't always know what you're gonna do. A: Mhm. P” And I guess that's sort of the part that we have to kind of figure out is me giving you some freedom but you also understanding that I need some information because I don't want, y'know I don't wanna get stressed out. A: Yeah.
No point anymore and minimal responses	Dyad members see no point in discussing a topic further. Characterized by expressed feelings of hopelessness about agreement. Dialogue slows, responses trickle to a minimum, one youth described “zoning out.” However, this does not mean either member has conceded. Tension: Endure or feint. Enduring dyads remain present but refuse concession or submission using minimal responses. Members sometimes feign cooperation or agreement or simulate flexibility without intention to negotiate or concede.	A: And I don't think I'll do um [water polo] in January because it was very stressful. I'll probably just do the [name of extracurricular sports team]. P: Well we'll have to see how that works out, see how your course load is and how everything else is going. A: Yeah and… P: Just have to wait and see. A: and, and I'll do some volleyball and basketball. P: There's lots of things we don't know about, just depends how um, how you find the work level and mm.

A review of the case summaries suggests that some dyads stayed with one theme and others appeared to include additional themes over the course of their conversation. Table 4 summarizes the variability in how many dyads fit different themes for the 23 conversations where there was at least one sequence.

**Table 4:** Count of different face‐to‐face resistance themes present in conversations.

Number of different themes	Count within dyad	Percentage (%)
1	5	21.74
2	7	30.43
3	10	43.48
4	1	4.35
Total	23	100.00

Table 4 shows the count of how many of the conversations contained from one to four of the four face‐to‐face resistance themes (e.g., the first row shows that five of the conversations contained only one of the themes over the course of the conversation). One pattern that emerged in the findings was that cases appeared to vary in the number of different themes in their conversations. Counts indicated that most cases (78.56%) were found to use more than one theme of face‐to‐face resistance over the course of their conversation. Dyads varied in the number of resistance sequences present in their conversation (range = 0−9), but each sequence reflected mainly one of the four developed themes. In terms of order, no resistance sequence regularly came before another sequence. It was rare for resistance sequences to end in agreement or submission (*n* = 24, 24.49%). Instead, most differences gradually and slowly came to a trickling of face‐to‐face resistance wherein both members struggled with a tension between two competing forces such as examining a disputed topic in detail or trivializing its importance. The following example of trickling came from a mother‐daughter dyad differing on whether the daughter take French:

P: Oh interesting, well that'll be another big transition then, do you not take one of your electives because of that?

A: No *not French*

P: Oh not French, I see OK but when would you take French?

A: Never

P: Well you need it to graduate though don't you?

A: No

P: You have to have a language

A: Mnm, you only have to have math and science and LA and one more

P: Hm, well, ok …

A: but I can take, yeah never mind

P: Ok well y'know the other transition is you'll be staying at school every day for lunch instead of going home

The disagreement in this example regarding the need to take French came to a bottleneck wherein neither member offered any more to working through the difference. They did not come to an agreement, and though not necessarily rejecting engaging with the other entirely, neither member was eager to jump into the conversation further. Both mother and daughter described similar feelings during video recall interviews ‐ that they were trying to get the other to listen.

## 5 Reminding of and then Defending the “Constant Battle” Lines

This theme was characterized by old boundaries or expectations being re‐raised and defended by parents and adolescents. Dyad members took a more hard‐line approach to resistance by cajoling, over‐talking, demanding, and making bottom line statements. One mother, for example, told the researchers during recall about maintaining house rules that had stood since her first child, saying “all the badgering and whining isn't going to change them.” We borrowed from hockey to call the tension associated with this theme slap‐shotting and blocking, with both partners simultaneously taking slaps and blocking each other's shots. The goal of some parents who were taking slapshots was to vigorously remind the other of expectations like maintaining good grades, participating in extracurricular activities, and not “frivolously wasting time” hanging out with friends. Parental expectations to balance multiple responsibilities could at times be overwhelming and as might be expected, these actions would sometimes be less than well‐received by some youth who described feeling forced or trapped during recall. Blocking strategies were sometimes direct and included protesting, arguing, exclaiming, and responding in determination (“So what? I can do it.”) However, strategies also took less direct forms like offering imprecise information, denying, stalling, or not responding at all. One youth responded to being pressed by blocking their parent's attempts to obtain information about going to a mall with friends. The youth gave the parent an intentionally imprecise answer “Any mall” or refused to provide information to the parent “Mm, none of your business.” Another youth responded to his mother's questions by telling her repeatedly that he did not want to attend his swimming classes; however, during recall he said, “I don't mind swimming, I just wanna get out of the conversation.” This same youth later explained he wanted to escape because he felt like “All I can do is say like, yes, I know.” Blocking was skillful for resisters who were able to find creative ways to maintain a sense of autonomy without disengaging from the conversation entirely.

Blocking strategies were observed to be both verbal and non‐verbal. In the following example, one youth blocked by stalling giving into his mother's demands. In the video, this youth can be seen lowering his head between his knees in his chair, responding minimally, and keeping his eyes trained to the floor. He went from minimal responses to eventually refusing to respond to his mother at all, changing the topic when his mother continued to press the issue:

A: I know, ‘cos I don't like writing stories.

P: You're not writing stories mm, in high school you do lots of writing, if you don't like it, you'll just dislike it more and more. You just need to get used to writing more.

A: OK.

P: And you can be better … …

A: … [noises of fidgeting]

P: (says shortened version of youth's name to get his attention)?

A: What?

P: You have to do work…

A: Sometimes.

P: It's easy [slight laugh]

A: [noises of fidgeting] Why's the camera still looking?

By refusing to respond, this youth blocked his mother's attempts to press him for information and leveraged his stalling technique to hold out for a better offer, ultimately achieving his goal of moving his mother's expectations for him. In the sense that this youth was able to achieve his goal of reaching a better offer without the mother enforcing a bottom line, this youth demonstrated skillful face‐to‐face resistance.

Finally, we observed an interesting pattern which occurred close to the end of some conversations when it came time for the parent and youth to get the researchers. Some parents and youth engaged in what one parent called a “constant battle of sort of a power struggle” where a request would be made by one member to go and get the researchers, and instead of refusing outright, the request would be reversed by the other (“Do you wanna?”) The parent and youth would go back and forth trying to get the other to concede but there appeared to be a threshold at which at least one member decided that it was no longer worth their time and energy to continue to resist, and they gave in. One parent said during the video recall interview “It's just too much trouble, I tend to give in.” One youth expressed during her recall “No matter what argument you get into, she always seems to win.” It was not uncommon at the tail end of such interactions for the member giving into make a jabbing remark as they capitulated (A: Yeah, I'm too lazy; P: Oh I know). This pattern may have been positive in that dyad members appeared to be making wise decisions about the extent to which yielding may have been the path that did not make the conversation any more uncomfortable.

## 6 Cautious Avoidance

Cautious avoidance was characterized by the dyad treading carefully around a sensitive topic (i.e., upsetting or a cause of concern) that was potentially contentious and likely to have been discussed in the past. Dyads treaded carefully around the topic to not cause a disturbance and maintain a sense of peace. Cautiously avoiding dyads managed what we chose to call a tension between avoiding (“Do I get to go home now?”) or approaching (“And the big thing for me is…”) a sensitive issue. In the following example, one dyad stepped carefully around the daughter's desire for more freedom:

P: So where do you wanna start (youth's name)?

A: I don't care.

P: You don't care? Do you wanna go for the gusto on the big issue first? Or do you want to pussy foot around some little issues?

A: Pussy foot.

P: Pussy foot. OK how d'you think you, how d'you think high school's gonna change our relationship?

A: [whispers] Our relationship? Um… I think I will get away more.

P: “Cos you want more freedom.”

A: Yeah, so I can like be away from the house a lot more. I hope.

P: OK.

A: “Cos our house isn't that big and it's not that fun.”

P: Well/

A:/So y'know

One youth from another dyad tried avoiding early in the conversation by quickly changing topics by slipping in a distraction of telling his mother about a movie he had recently watched (“I just wanna tell you.”) During this youth's recall, he said he had done this to see if he could “throw off” the conversation by teasing his mother in an endearing way and having fun together. On the other hand, approaching strategies included bracing the other by starting with a compliment, using humor to act as a buffer for the relationship, or using rounding phrases (“That's all I'm saying, that's all.”) to temper or soften the impact of one's words. One youth who wanted her mother to purchase a new cell phone for her approached the topic gingerly using laughter and humor to hint at her dislike of her current phone.

Dyads that struggled to resolve the tension in this theme sometimes signaled their desire to discontinue cautious avoidance by trying to change topics to which the other could concur, thereby, terminating the discussion. For example, one parent abruptly changed topics to avoid having to openly acknowledge that she knew about her son's substance use (“Ok, moving on.”). During recall, the mother said that she was aware that her son was using substances, and the son in his recall said he was aware that his mother knew he was using. However, the mother carefully avoided open discussion about it because, according to her recall, she felt uncomfortable about the prospect of her son ignoring what she says and having to get into a “huge struggle” with him. It appears that face‐to‐face resistance may be viewed as adaptive in so far as such patterns help dyads to defer having conversations that could be unpleasant.

## 7 The Nudging Match

Parents and youths would sometimes get into perception disputes over how important a topic was and whether continuing to discuss it was necessary. We called this tension shrinking (“Why does it matter?”) and magnifying (“But what if…”) Magnification involved calling the other's attention to a topic's importance by pressing them to contemplate it in detail. For example, some dyad members used hypothetical situations to encourage the other to plan contingencies for specific circumstances (e.g., planning how not to get into a car with someone who has been drinking or thinking about how to respond when getting asked on a date). One parent said during the video recall interview that she used a hypothetical situation to resist her daughter's tendency of having “an answer for everything,” pushing her daughter to think deeper by suggesting different possible contingencies. One parent explained during recall that he used hypothetical situations to get his daughter to expand her confidence and competence in handling future dilemmas because “she closes off to that reality.” On the other hand, shrinking involved downplaying the significance of a topic (“She doesn't have to be worried,” “calm down”), especially if the dyad had discussed the topic in the past. Alternatively, shrinking involved drawing the others' attention away from a topic. For example, some of the youth engaged in shrinking by diverting their parents' attention by making unrelated observations or playful non‐sequiturs (e.g., “Do you know there's a camera in here?” “I haven't had my juice today.”)

The goal of magnifying and shrinking appeared to be to try to move or nudge the other away from how they currently perceive a topic but in a way that would circumvent a full‐blown confrontation. This nudging tended to be in the service of a goal one would like to achieve from the other. Parents or youth would sometimes try to bring the other's perspective on a topic in line with their own so that they could obtain a desirable outcome from their partner's actions. A common example was for parents to magnify how high school could be less‐than‐forgiving with homework reminders to encourage the use of planners in their adolescents. The overall parental goal was for their adolescents to see the importance of being organized through magnification of high school as less “hands on” with students than elementary school. Another example involved one youth who engaged in magnifying wanting permission from his guardian to learn hunting by venerating it, calling it “the art of hunting” and tried to pre‐empt objection by describing it to her as something he knew she would never let him do. The mother engaged in shrinking by deflecting to laws around shooting age, deferring agreement by telling the youth “We'll see” and abruptly changing topics. Some youth and parents alike explained in their recall interviews that how a topic or person is perceived matters because perceptions play a significant role in guiding future actions. One youth, for example, said it was important to her that her mother saw her as trying in school despite her recent poor academic performance. She explained that if her mother perceived that she was not trying in school, she would not be allowed to go out with friends. Thus, the daughter repeated at several points in the conversation that she was trying in school, telling her mother “It's not like I don't try because I do, I do.”

One mother described the magnifying and shrinking tension as “a dance that we do” which involved her pressing for information for details of her daughter's plans using questions in quickfire succession, and the other providing minimal or imprecise answers, which led to more questions from the mother. During recall, this mother commented on the resistance sequence below by mimicking an example of “the dance”: “‘Well, where are you going?’ ‘I don't know,’ ‘How are you gonna get there?’ ‘We're not sure yet’.”

P: Yeah like if I don't know exactly where you are and when I ask you just sort of say you y'know [funny voice] “I don't know we're just gonna hang out.” [slight laugh]

A: But I don't know so that's why I say it.

P: I know and that's true, you don't always know what you're gonna do.

A: Mhm.

P: And I guess that's sort of the part that we have to kind of figure out is me giving you some freedom but you also understanding that I need some information because I don't want, y'know I don't wanna get stressed out.

A: Yeah.

P: Right? And you're also entering that age where, where your friends are y'know it won't be that long before you have friends that have driver's licenses right and that.

The mother said in her recall, “I'm not sure she's telling me everything… and I don't want to push it ‘cos I don't want to back her into a corner where she has to feel defensive’.” One youth explained during recall that she was reviewing all the details of her plans in her head during her conversation with her mother in anticipation of the questions her mother might ask, understanding that not having satisfactory responses could lead to “a big mumbo jumbo” of having to face more questions and possibly being denied permission to spend time with her friends. Some parents described during recall their awareness of a balance between pressing for information and a tipping point at which their efforts might be perceived as overbearing. An important aspect of the nudging match occurred when a dyad member would resist the conversation that the other would attempt to engage in (i.e., avoiding the “big mumbo jumbo”) by refusing to “take the bait” of their partner using their resistance strategies. Face‐to‐face resistance thus appeared to require a degree of tact that when employed skillfully helped dyad members achieve their goals in a way that did not lead to being perceived in a negative way.

## 8 No Point Anymore and Minimal Responses

The endgame of face‐to‐face resistance tended to occur when dyad members saw no point in discussing a topic further. This theme was characterized by expressed feelings of resignation or hopelessness during recall about coming to an agreement together and the realization that their attempts at nudging had been unsuccessful. Said one youth during recall “I just get tired of arguing, argument like these questions.” One youth described during the video recall interview a point at which she started “zoning out” when being questioned by her mother. Dialogue tended to slow, and responses became minimal once face‐to‐face resistance had reached this point. However, this did not mean that either member had conceded. Instead, recall interviews revealed that some dyad members overrode or expressed their intent to proceed with their plans regardless of the other's disapproval. In the following example, one mother stated during recall her intent to continue to keep her daughter enrolled in multiple extracurricular sports “whether she likes it or not” based on the belief that participation would keep her daughter out of trouble:

A: And I don't think I'll do um [water polo] in January because it was very stressful I'll probably just do the [name of extracurricular sports team].

P: Well we'll have to see how that works out, see how your course load is and how everything else is going.

A: Yeah and…

P: just have to wait and see.

A: and, and I'll do some volleyball and basketball.

P: There's lots of things we don't know about, just depends how um, how you find the work level and mm.

A: Yeah.

P: everything. Right?

A: Yeah.

Based on such examples, we called the tension to be navigated for dyads who saw no point as endure or feint. Enduring was different from dis‐engagement in that the dyad members remained present with each other but refused to concede or submit using minimal responses. Some parents and youth feinted cooperation or agreement or simulated being flexible without intention to negotiate or concede. One example of this feint was of a mother who wanted her son to attend a school she had selected against her son's wishes; the son wanted to attend a school of his choice and she promised to keep his choice open during the conversation. Feinting did not usually arouse an aversive reaction from a partner and did not require submission; however, as in boxing, it was a way to avoid requests or demands coming from a partner. Dyad members whose interactions were characterized by this theme directed their efforts, paradoxically, towards not having to make extra efforts. They no longer wanted to explain themselves or push back explicitly but were still focused on their goal, not necessarily needing involvement or buy‐in from the other. Individual goals had not been killed, they just did not involve input from another, making interactions potentially superficial. Face‐to‐face resistance was positive in this tension in that dyad members chose actions that would not lead to a conversation that may have resulted in potentially heightened negative emotions in themselves or in their partner. By appearing to give in, a dyad member precluded any further retort from their partner, thereby sparing themselves from having to delve further into a potentially contentious topic during the conversation.

## 9 Discussion

The aim of this study was to describe face‐to‐face resistance patterns in parent‐adolescent conversations about the upcoming transition to high school. Our main findings were the development of four face‐to‐face resistance process themes. Case summaries suggested that dyads engage in resistance in their own distinct ways. That is, dyads differed in how they perceived topics, expressed, and navigated their differences. Moreover, there was considerable variation between dyads when it came to how they navigated their differences together. Adopting a dyad‐centric, moment‐to‐moment approach to analysis was useful to be able to see such differences. We suggest that taking such an approach was important because it may have been more sensitive in capturing the nuances of the dyad‐centric ways of engaging together in resistance that could reflect a personalized relationship history. To the best of our knowledge, this was the first study to treat resistance between parents and youth as a dyadic concept and attempted to describe how dyads engage in navigating their differences together during everyday interactions.

## 10 Implications for Research and Theory

One contribution of this study's findings to the body of resistance research was that rather than ending in agreement, many resistance sequences gradually slowed to a trickle. Some dyad members reached a point near the end of sequences where they disagreed and were neither fully engaged nor disengaged, with one member eventually flipping to a new topic. For example, a dyad member could change topics abruptly, employ generic phrases (e.g., “we'll see,” “so that's it,” “yeah, maybe”) or choose to stall or not respond. One implication of trickling is that some parent‐adolescent disagreements may remain superficial. Dyads where trickling was present often moved away from disagreements rather than approaching them, sometimes to maintain a sense of peace and defer discussing topics that were sensitive or painful. If this pattern of non‐addressing continues then the disagreement may come to a head in the future in the form of an intense conflict, or they may end up not addressing a topic at all. The consequences to the parent‐adolescent relationship of trickling have yet to be studied.

The observation of trickling has led us to propose that there are dyadic‐level processes that occur during parent‐adolescent disputes that do not map easily onto how conflict has been conceptualized and operationalized. Parent‐adolescent conflict resolution research has tended to divide resolution into adaptive or disruptive strategies (Branje et al. 2009; Ferrar et al. 2020; Moed et al. 2015) associated with adaptive (Missotten, Luyckx, Branje, et al. 2017; van Doorn, Branje, and Meeus 2011) or maladaptive outcomes (Huey et al. 2017; Flamant et al. 2020). These studies tended to focus on the management of conflict. Trickling, however, was a uniquely dyadic phenomenon within face‐to‐face resistance that was difficult to judge as either adaptive or maladaptive. Within the broader literature, trickling serves as an example of the types of microprocesses that could be captured when investigating resistance beyond individual strategies. Further investigation into dyadic resistance microprocesses may elucidate the many ways parent‐adolescent relationships can evolve to take distinct shapes in families with adolescents.

The patterns of face‐to‐face resistance developed in this study may contribute to an existing body of literature that conceptualizes interpersonal influence as relationship‐dependent. Learning to navigate differences together (especially during significant transitions) is an important process parents and adolescents engage in that can bear on the quality of the parent‐adolescent relationship. We interpreted the care with which parents and adolescents broached or avoided discussing potentially sensitive topics during video recall interviews as an indicator of tact that warrants further attention. Future studies may examine whether and why some parents and adolescents are likely to remind of and defend battle lines and others engage in nudging. Researchers interested in parent‐adolescent relationships may find the face to face patterns described in this study helpful for advancing understanding of the microprocesses involved when parents and adolescents engage in disputes. For example, there are opportunities to probe further whether and how some dyads move from nudging to minimal responses, or the likelihood of dyads transitioning from defending battle lines to cautious avoidance. We were unable to assess whether there are patterns of resistance that are correlated or tend to follow sequences across time, nor could we specify whether some topics are likely to be associated with certain resistance patterns. We suggest that the patterns from this study may aid in the development of a more fulsome understanding of the context‐dependent ways disputes can unfold.

Rather than being viewed as uniquely adaptive or maladaptive, adolescent development researchers have emphasized that it is important to investigate how conflicts unfold and how young people manage conflict with parents (Ferrar et al. 2020; Missotten, Luyckx, Vanhalst, et al. 2017). Putting this study's findings in the context of research on parent‐adolescent conflict, we propose that conflict measures may be missing important information on what happens when parents and adolescents disagree. Collins and Laursen (1992) have argued that measures of conflict must be sufficiently sensitive to provide uncontaminated information about the variability of disagreements or oppositions across both dyads and time. However, studies using conflict measures like the Issues Checklist (Prinz et al. 1979) have tended to yield consistently low rates of conflict incidence (Denuwelaere and Bracke 2007; Smetana and Gaines 1999) even though low‐level disputes are reportedly common in adolescence (Weymouth et al. 2016).

By comparison, we observed differences in viewpoints occurring at least once in over 85% of conversations but differences did not necessarily become intense. Therefore, current conflict measures might not be able to see such differences because dyads regularly resist, slip, move away from, or find ways to avoid full‐blown confrontations. However, we suggest that understanding what happens when dyads resist is important because it could have implications for both parents' and adolescents' identity construction and evolution (Grotevant and Cooper 1985; Hauser et al.^1984^).

The process of navigating differences in viewpoints may provide opportunities for adolescents to construct a sense of self that is part of, yet distinct from their parents. The idea that parent‐adolescent conflict can play a functional role in adolescent autonomy development is not new to researchers of adolescence (Adams and Laursen 2007; Cooper 1988; Hill and Holmbeck 1987; Laursen and Collins 2009). However, conflict in families is different from conflict in other types of relationships in that families must manage and work through their differences together and are less able to walk away from each other. Family conflict theory sees conflict as a dialectical process of managing a perpetual tension in intimate relationships between the need for autonomy and the need for togetherness (Sprey 1979; White, Martin, and Adamsons 2019). This dialectic‐like process is similar to Adams and Marshall's (1996) developmental social psychology of identity which posited that individuals need both differentiation and integration. Individuals strive to balance the process of constructing a unique self and the process of connecting with and belonging to others within a relationship context (Adams and Marshall 1996). Balance between these processes of interpersonal differentiation and integration has been argued to be critical for healthy growth (Grotevant and Cooper 1985). A difference may represent a form of incompatibility or distress that can give rise to the need for change, the navigation of which can lead to synthesis. The dialectical model of intergenerational transmission depicts parents and adolescents as engaging in a transactional process of facing contradictions that emerge from different perspectives within the constraining influence of an interdependent relationship (Kuczynski and De Mol 2015). Dyads may reconcile different sources of information from their micro‐level interactions to reconstruct or affirm ideas of how to interact with each other in potentially novel ways. Thus, back‐and‐forth face‐to‐face resistance may have been when some adolescents and their parents were both constructing a dynamic sense of who they are within the context of their ever‐changing relationship.

Young people are not the only ones who experience significant changes during a family's transition to adolescence. Adolescence can coincide with midlife, a time of significant physical and emotional adaptation for parents. Parents in midlife may start to experience the cognitive and physical effects of aging (Lachman 2004) and increases in parenting stress if they have dysfunctional interactions with their children (Kochanova, Pittman, and Pabis 2021; Putnick et al. 2010). Moreover, parenting stress has been known to be a salient factor in the well‐being of many parents of adolescents (Arbel et al. 2020). Mothers have been found, for example, to be more sensitive and strongly affected by negative parent‐child conflicts interactions than adolescents (Missotten, Luyckx, Vanhalst, et al. 2017). On the other hand, mothers and adolescents both have been found to benefit from interaction patterns characterized by mutual attempts to handle conflict in a constructive way (Missotten, Luyckx, Branje, et al. 2017). Being able to handle differences positively may promote parental well‐being and feelings of positivity about the relationship, which may reaffirm that they are being effective in their parental role. From this point of view, interactions like face‐to‐face resistance are healthy for individual growth as well as for the progressive and mutual adaptation of the parent‐child relationship. What practitioners may be seeing in their encounters with struggling parent and adolescents may be escalation rooted in being stuck in ways of navigating differences that are not facilitating synthesis or agreement for them.

The four themes presented in this study may suggest that it is possible to identify patterns of resistance that occur with regularity over time across dyads. There might be opportunities in the future to study trajectories of resistance during interactions to predict whether there are ways of going back and forth that are associated with or are more likely to lead to other ways of resisting that follow in time. For example, it may be possible to map the trajectory of resistance across the duration of parent‐adolescent conversations to describe what face‐to‐face resistance looks like at the dyadic level of analysis moment‐to‐moment. Such analyses might show that there could be resistance attractors, how resistance tends to start, and common destinations or end points in resistance sequences. The next steps may be to see how possible trajectories mapped onto a grid relate to new data collected from parent‐adolescent dyads from different cultural contexts, or at different ages across adolescence.

### 10.1 Limitations

Even though the applicability of findings to diverse cultural backgrounds is limited, the significance of the influence of cultural contexts in shaping parent‐child relationships and conflict dynamics cannot be overstated. Parent‐adolescent conflict has been found to vary depending on a dyad's national origin in Hispanic families (Li and Warner 2015). Differences have been found between Chilean, Filipino, and American adolescents in the extent to which young people feel the need to obey their parents across different social domains (Darling et al. 2006). Parent‐adolescent conflict has been found to predict lower levels of emotional and physical comfort as well as poorer home health and safety in high‐risk, inner‐city African American 14‐year‐olds (Griggs et al. 2019). Such studies highlight the influence of cultural norms, socialization practices, and core values on parent‐adolescent interactions. Important cultural nuances or ways of expressing and navigating conflict specific to home languages may not have been fully captured or accurately represented due to the interactions being in English. Not being able to communicate in their primary language means that for the five dyads who spoke languages in addition to English at home, representation of resistance might have been partial and not reflective of true interaction dynamics of these dyads.

The nuances amongst individuals with seemingly similar cultural backgrounds were unaddressed due to the use of broad cultural categories. For example, “Asian” encompasses a wide range of cultural groups and subgroups, each with their own distinct ways of parenting, cultural expectations and concepts, and acceptable ways of navigating disagreements that were not parceled out and analyzed in this study.

Collecting data in a university setting wherein dyads were videorecorded may have introduced certain limitations to this study. First, the awareness of being recorded may have made dyad members temper their behaviors more than if they were in a naturalistic setting like in the home, trying to make their responses appear as socially desirable as possible. Another limitation of being recorded in a room could be that dyad members would be less compelled to simply walk away from a conversation. Another limitation of this research was that it was cross‐sectional. Studies using longitudinal designs could add value to our evolving understanding on the stability or variability of face‐to‐face resistance patterns. For instance, analysis of longitudinal data could reveal whether face‐to‐face resistance changes over time or is inextricably linked to transitions like the transition to high school. The small sample analyzed in this data consisted of Canadian dyads of parents and adolescents discussing the upcoming transition to high school. Given that our sample was predominantly English speaking, not Francophone, and from European background, the degree to which the results may generalize within Canada and across cultures may be limited. It is also important to acknowledge that the data analyzed for this study were collected 14 years ago. Significant societal, cultural, and technology‐related shifts may influence the relevance of the findings to parent‐adolescent dyads today. Finally, the small number of fathers participating in the research limits understanding of how gendered familial roles influence resistance patterns. Thus, more research is needed to explore whether the themes developed in this study are relevant to groups across multiple areas of diversity.

### 10.2 Strengths

One strength of this study is the introduction of an original method to qualitatively analyze dyadic data. Its development was inspired by a growing interest in the field of parent‐adolescent relationships on what dyads do during their moment‐to‐moment interactions. Another strength is that the data analyzed were observational, which is useful for observing micro‐level moment‐to‐moment interactions (Gottman and Notarius 2000). Observational data are beneficial because parents and youth who self‐report have been found to be more likely to endorse constructive resolutions than they practice during actual conflict situations (Collins and Laursen 1992). Another benefit of this study was that data were both multi‐source (from parents and adolescents) and multi‐informant (video recall as well as conversational data). Having access to video recall interviews aided interpretation of participant's meaning and intent during sequences.

## 11 Conclusion

To date, resistance has been investigated as an individual behavior (either teen resistance or parent resistance). This study offered insight into how face‐to‐face resistance unfolds as micro‐processes during conversations about the upcoming transition to high school in a sample of Canadian parent‐adolescent dyads. We examined face‐to‐face resistance in parents and adolescents using data comprised of video‐recorded self‐directed conversations with separate video‐recall interviews. A secondary analysis was conducted on the data using a variant of critical reflexive thematic analysis guided by social constructionist theory at the dyadic level of analysis. Our findings offered evidence of resistance as a dyadic concept wherein both partners contribute creating non‐independence during interactions, with considerable cross‐dyad variability in terms of timing of first occurrence, how often dyads engaged in resistance during conversations, and how many different themes were present during conversations. One new phenomenon we observed was that of trickling, wherein some members did not agree, but also did not reject engaging with their partner entirely, with neither member extending the resistance further. A dyad‐centric, moment‐to‐moment approach in future research may be more sensitive at capturing the nuances of how parents and adolescents engage in resistance together that could reflect a personalized relationship history. Research on the specific mechanisms that occur during everyday parent‐adolescent interactions could contribute to our understanding of how relationships evolve and may have implications for the development of the identity of parents and their adolescent children.

## Ethics Statement

All co‐authors have seen and agree with the contents of the manuscript and there is no financial interest to report. We certify that the submission is original work and is not under review at any other publication.

## Conflicts of Interest

The authors declare no conflicts of interest.

## Data Availability

Data sharing is not applicable to this study as no new data were generated for this study. Data are not shareable due to privacy restrictions.
